# Nutritional Risk Score (NRS-2002) as a Predictor of In-Hospital Mortality in COVID-19 Patients: A Retrospective Single-Center Cohort Study

**DOI:** 10.3390/nu17071278

**Published:** 2025-04-06

**Authors:** Jan Ilkowski, Przemysław Guzik, Aleksandra Kaluźniak-Szymanowska, Piotr Rzymski, Jerzy Chudek, Katarzyna Wieczorowska-Tobis

**Affiliations:** 1 Department of Emergency Medicine, Poznan University of Medical Sciences, 61-701 Poznan, Poland; janilk@onet.eu; 2 Department of Cardiology—Intensive Therapy, Poznan University of Medical Sciences, 61-701 Poznan, Poland; pguzik@ump.edu.pl; 3 University Centre for Sports and Medical Studies, Poznan University of Medical Sciences, 61-701 Poznan, Poland; 4 Geriatric Unit, Departement of Palliative Medicine, Poznan University of Medical Sciences, 61-701 Poznan, Poland; kwt@tobis.pl; 5 Department of Environmental Medicine, Poznan University of Medical Sciences, 60-806 Poznan, Poland; rzymskipiotr@ump.edu.pl; 6 Department of Internal Medicine and Oncological Chemotherapy, Medical Faculty in Katowice, Medical University of Silesia, 40-055 Katowice, Poland; chj@poczta.fm; 7 Department of Human Nutrition and Dietetics, Poznan University of Life Sciences, 60-637 Poznan, Poland

**Keywords:** COVID-19, in-hospital mortality, malnutrition, Nutritional Risk Score (NRS-2002), risk assessment

## Abstract

**Background**: Malnutrition is an often-overlooked yet potentially crucial factor influencing COVID-19 outcomes. Poor nutritional status weakens immune function, increases infection susceptibility, and worsens prognoses in hospitalized patients. However, its specific role in COVID-19 mortality remains insufficiently characterized. The aim of the study was to assess the impact of malnutrition, as determined by the Nutritional Risk Score (NRS-2002), on in-hospital mortality. **Methods**: This retrospective, single-center study analyzed 222 patients hospitalized with COVID-19 during the Delta variant predominance. Thirty-one patients died during hospitalization. Malnutrition (NRS ≥ 3) emerged as a strong predictor of in-hospital mortality in univariate Cox proportional hazard models, both before and after adjustment for potential confounders. Adjusted analyses used 10 different sets of three out of five mortality-related variables. **Results**: Hazard ratios for malnutrition ranged from 3.19 to 5.88 (*p* < 0.01 for all models), highlighting its substantial impact on mortality risk. The high Nagelkerke’s R^2^ values (0.66–0.77) indicate that the models explained a significant proportion of mortality variance. Nutritional status plays a critical role in COVID-19 survival among hospitalized patients. **Conclusions**: Given its simplicity and effectiveness, integrating the NRS-2002 into routine clinical assessments may help identify high-risk patients early. Future research should explore whether early nutritional interventions can mitigate the mortality risks associated with malnutrition in severe COVID-19 cases or patients with other infectious diseases or acute inflammation.

## 1. Introduction

The COVID-19 pandemic posed unprecedented challenges to healthcare systems worldwide, with a significant focus on identifying risk factors for severe outcomes. While age, comorbidities, and immune dysfunction have been widely recognized as determinants of COVID-19 severity [1,2,3], the role of nutritional status remains inadequately characterized. However, it was shown that malnutrition is a common problem in hospitalized older individuals due to COVID-19, with the frequency ranging from 41.1% to 85.8% depending on the diagnostic criteria adopted and the severity of the disease [4,5].

The studies exploring the associations between dietary patterns and COVID-19 severity highlight that plant-based, Mediterranean, and Dietary Approaches to Stop Hypertension (DASH) diets are linked with favorable outcomes. In contrast, a diet rich in processed foods, saturated fats, sugars, and additives is associated with a higher risk of severe disease [6]. On the other hand, poor nutritional status in patients hospitalized due to COVID-19 was associated with negative outcomes. Larrázabal et al. [5] found that malnutrition correlated with prolonged hospital stays and extended mechanical ventilation. Similarly, meta-analyses by Boaz et al. [7] and Abate et al. [8] linked malnutrition to a higher risk of ICU (Intensive Care Unit) admission rate and in-hospital mortality. However, conflicting findings exist—Bedock et al. [9] reported no significant association between nutritional status and COVID-19 severity, while Sánchez-Rodriguez et al. [10] found no link between malnutrition and mortality in older adults hospitalized with COVID-19. Nevertheless, a meta-analysis by Feng et al. [11] underscored the high prevalence of malnutrition risk in this population, advocating for routine screening and nutritional support. However, further validation is needed across diverse clinical settings to confirm these findings. Beyond individual outcomes, addressing malnutrition has broader public health implications. As a widespread issue affecting low- and high-income countries, nutritional disparities may exacerbate COVID-19 severity and future pandemic resilience.

Various validated screening tools can be employed to assess malnutrition risk in patients hospitalized with COVID-19, including the Malnutrition Universal Screening Tool (MUST) [12,13], Controlling Nutritional Status (CONUT) [14], the modified Nutrition Risk in the Critically ill (mNUTRIC) [15], Geriatric Nutritional Risk Index (GNRI) [16], and the Nutritional Risk Score 2002 (NRS-2002) [17]. The latter, NRS-2002 screening, was found to be among those detecting the highest prevalence of malnutrition [8] and had a higher area under the curve value than other tools in predicting in-hospital mortality among COVID-19 patients, indicating its robust predictive capabilities [17].

This study aimed to investigate whether NRS-2002 independently predicts in-hospital mortality in COVID-19 patients during the Delta variant predominance. The Delta SARS-CoV-2 variant (B.1.617.2), predominant from mid-2021 to early 2022 [18], was associated with heightened transmissibility, increased viral loads, and greater hospitalization risk [19,20]. The studies also evidenced that patients hospitalized during the Delta-dominated period were younger, less comorbid, had the highest need for oxygen support and mechanical ventilation, and risk of death [1]. The rapid progression of infection to hypoxemia emphasized the need for reliable prognostic tools beyond age and comorbidities, particularly in resource-constrained settings.

We hypothesized that higher NRS-2002 scores will increase mortality, even after adjusting for age, comorbidities, and disease severity. By analyzing a single-center cohort during this critical period, we aim to provide evidence for the role of malnutrition as a modifiable risk factor and support using NRS-2002 as a prognostic tool. Our study builds upon prior research by specifically examining the role of malnutrition during the Delta variant wave, a period characterized by increased severity and hospitalization rates. While previous studies have identified malnutrition as a factor in COVID-19 outcomes, our analysis applies robust statistical modeling to demonstrate the independent predictive value of NRS-2002. By adjusting for key confounders and testing multiple models, we strengthen the argument that malnutrition screening should be systematically integrated into routine clinical assessments for hospitalized COVID-19 patients. This study also highlights the broader implications of malnutrition screening in acute infectious diseases, reinforcing the importance of nutritional interventions beyond the COVID-19 context.

## 2. Materials and Methods

### 2.1. Study Design

This is a retrospective data analysis of patients hospitalized in the Neurology Ward of the Hospital of the Ministry of the Interior and Administration in Poznan, Poland, due to COVID-19. This ward was transferred into a monomial COVID-19-dedicated unit on September 10, 2021; thus, all hospitalized patients had positive results of RT-PCR tests for SARS-CoV-2. Admission decisions were made following triage by experienced internal medicine specialists, considering the clinical presentation and laboratory and imaging results. Patients who required mechanical ventilation due to respiratory failure were directly transferred to ICU, not to our ward. We analyzed patients hospitalized until 15 January 2022, as the Delta variant of the COVID-19 virus was dominant at that time [18].

Due to the presented study’s retrospective nature, participants’ written consent was not required. The presented project was approved by the Bioethical Committee of the Poznan University of Medical Sciences (approval number 550/24) and performed according to the ethical standards laid down in the 1964 Declaration of Helsinki and its later amendments.

### 2.2. Patients

There were 225 patients hospitalized in the Neurology Ward in the analyzed period. Three patients were not included in the analyses, as they had disseminated metastatic cancer at the end of life. Thus, 222 patients’ data were analyzed in this study.

### 2.3. Data Collection

For all patients, demographic information (age, sex), clinical data, comorbidities, selected laboratory parameters, treatment, and outcome data (discharged or dead during hospitalization) were extracted from the in-hospital medical records. We aimed to minimize information bias by cross-verifying data sources.

The medical history of cardiovascular diseases (hypertension, congestive heart failure, ischemic heart disease, arrhythmias, and valvular diseases), chronic obstructive pulmonary disease (COPD) or asthma, diabetes, kidney failure, stroke/TIA, and neurodegenerative diseases was also retrieved. Moreover, vaccination status was also one of the parameters considered in the study.

As far as clinical data are concerned, the presence of cognitive impairment (including delirium) was taken into account. Cognitive impairment was determined based on standard clinical assessment procedures, including physical assessment by attending clinicians upon admission to the hospital, as well as past medical history assessment. Additionally, a routine check of the nutritional state was performed using the NRS-2002 (Nutritional Risk Score). This questionnaire assesses two key aspects of malnutrition: the deterioration of nutritional status, which includes factors such as weight loss, low BMI, reduced dietary intake, and increased nutritional requirements due to both acute and chronic illnesses. Additionally, individuals aged 70 years and older receive an extra point to account for age-related factors. The maximum possible score on the NRS-2002 is 6 points (7 for older adults), and a score of 3 points or more indicates a risk of malnutrition, signaling the need for timely nutritional intervention.

The Activities of Daily Living (ADL) were assessed by nursing staff using the Katz Index, which evaluates six basic functions: bathing, dressing, toileting, transferring, continence, and feeding [21]. Each function is scored as independent (1 point) or dependent (0 points), with the total score ranging from 0 to 6. A score of 4 or less indicates functional impairment. Furthermore, the risk of pressure sores was assessed using the Norton scale—a result below 14 points (20 points scale) implicates a greater risk of developing said sores. The scale includes the assessment of physical condition (good—4, fair—3, poor—2, very bad—1), mental condition (alert—4, apathetic—3, confused—2, stuporous—1), activity (ambulant—4, walks with help—3, chairbound—2, bedfast—1), mobility (full—4, slightly impaired—3, very limited—2, immobile—1), and incontinence (none—4, occasional—3, usually urinary—2, urinary and fecal—1).

Analyzed laboratory parameters included hemoglobin concentration, D-dimer level, white blood cell (WBC) count, and the concentration of other plasma inflammatory markers (C-reactive protein—CRP, procalcitonin—PCT). Anemia was defined as a hemoglobin concentration below the normal range and corrected by gender (<12 g/dL for females and <14 g/dL for males, according to the cut-off values in the hospital laboratory). Inflammatory markers were above reference when WBC count > 10^3^/μL, plasma PCT > 0.5 ng/mL, and CRP > 20 mg/L. The reference values for D-dimer were 0–0.5 g/mL.

The prescription of antiCOVID-19 therapy, antibiotics, and oral anticoagulants were analyzed. Non-invasive oxygen therapy was also taken into consideration.

The date of discharge or death defined the duration of the hospitalization of each patient.

### 2.4. Statistical Analysis

PQStat v.1.8.6.120 (PQStat Software, Poznań, Poland) was used for statistical analysis. Normality in the data distribution was examined using the Shapiro–Wilk test. Descriptive results are presented as medians, ranges, and 25th and 75th percentiles (Q1 and Q3, respectively). Categorical data were compared with Fisher’s exact test, and continuous data were compared with the Mann–Whitney test. Additionally, for numerical data, the Spearman rho coefficient measured correlations. A *p*-value of <0.05 was considered statistically significant.

Although this study utilized retrospectively collected data, the Cox proportional hazards model was employed to analyze in-hospital mortality in a longitudinal framework, estimating event probabilities over time during hospitalization. All mortality outcomes were ascertained from the hospital information system (HIS), ensuring complete capture of in-hospital events without needing prospective follow-up beyond discharge. For univariate adjusted Cox proportional hazard regression models, we included only the variables that significantly differed between survival and non-survival individuals in Fisher’s exact test. We conducted univariate adjusted Cox proportional hazard regression models to evaluate the association between malnutrition (Variable A) and mortality. Each model included malnutrition as the primary exposure variable and was adjusted for combinations of three additional covariates selected from five potential confounders (Covariates B, C, D, E, and F), all of which appeared to be associated with in-hospital mortality in the univariate unadjusted Cox proportional hazard models. All possible models were analyzed. This resulted in 10 models, each systematically adjusting for different combinations of these covariates. For each model, we reported the hazard ratio (HR), 95% confidence interval (CI), and *p*-value for malnutrition to assess its association with mortality. Additionally, we calculated Nagelkerke’s R^2^ and the corrected Akaike Information Criterion (AICc) to evaluate model fit and performance. This approach ensured that the relationship between malnutrition and mortality was robust to adjustments for potential confounders while adhering to statistical guidelines for event-per-variable ratios.

## 3. Results

### 3.1. Baseline Characteristics

The study included 222 patients (116 males, 52.2%) with a median age of 69.0 years (IQR: 57.0–81.0). Among them, 159 patients (71.6%) were at least 60 years old.

At hospital admission, 67 patients (30.2%) presented with cognitive impairment, including four younger individuals aged 25, 29, 30, and 39 years. According to the Norton scale, 81 patients (36.5%) were at high risk for pressure ulcers, while the NRS-2002 identified 58 patients (26.1%) as requiring nutritional support. Regarding functional status, 32 patients (14.4%) were partially dependent, and 84 (37.8%) were totally dependent on admission.

A history of cardiovascular disease was present in 140 patients (63.1%), including 118 (53.2%) with hypertension and 64 (28.8%) with other cardiovascular conditions. Additionally, 65 patients (29.3%) had diabetes, 32 (14.4%) had neurodegenerative diseases, and 38 (17.1%) had a history of stroke. Of note, only 45 patients were vaccinated against COVID-19.

Laboratory findings at admission are summarized in Table 1. Hemoglobin concentration was below the reference range in 77 patients (34.7%), while inflammatory markers were elevated in many cases. The median CRP concentration was 64.6 (IQR: 24.6–122.8) ng/dL, procalcitonin 0.12 (IQR: 0.07–0.3) ng/dL, and WBC count 8.6 (IQR: 6.6–11.9) cells ×10^3^/μL, with five patients presenting values below the reference range.

Strong negative correlations were observed between NRS-2002 scores and both the Norton scale (rho = −0.69, *p* < 0.001) and ADL scores at admission (rho = −0.70, *p* < 0.001). Moderate positive correlations were found between NRS-2002 and procalcitonin concentrations (rho = 0.46, *p* < 0.001), as well as NRS-2002 and CRP (rho = 0.35, *p* < 0.001).

### 3.2. In-Hospital Mortality Analysis

The median hospital stay was 11.0 (IQR: 9.0–14.0), with the longest duration being 31 days. A total of 31 patients (14.0%) died during hospitalization, of whom 25 (80.6%) succumbed within the first 14 days.

A comparison of survivors and non-survivors is presented in Table 1. Non-survivors were significantly older than survivors (*p* < 0.001) and had higher rates of malnutrition risk, pressure ulcer risk, and cognitive impairment (*p* < 0.001 for all). Poor ADL at admission was observed in 93.6% of non-survivors compared to 45.6% of survivors (*p* < 0.001).

The rates of comorbidities were similar in survivors and non-survivors, but the number of non-survivals with increased concentrations of selected inflammatory markers was higher (Table 1).

The factors influencing in-hospital mortality, comparing survivors and non-survivors, were analyzed using a univariable Cox proportional hazard model. The results are presented in Table 2.

The results of univariate Cox model evaluations demonstrate that the risk of malnutrition at admission is a consistent and significant predictor of in-hospital mortality across all ten models (Table 3), with hazard ratios (HR) ranging from 3.19 to 5.88 and *p*-values consistently below 0.01. HRs greater than 1 indicate high-risk patients. The 95% confidence intervals (CI) for the HRs do not cross 1, further confirming the robustness of this association. The models exhibit strong explanatory power, as indicated by Nagelkerke’s R^2^ values ranging from 0.66 to 0.77, suggesting that the included covariates explain a substantial proportion of the variance in mortality. Nagelkerke R^2^ ranges from 0 to 1, with values closer to 1 indicating a better fit of the model. Additionally, the corrected Akaike Information Criterion (AICc) values are comparable across all models, indicating a similar model fit. The *p*-values for the overall models are all highly significant (<0.001), further validating the statistical reliability of the findings.

**Figure 1:**
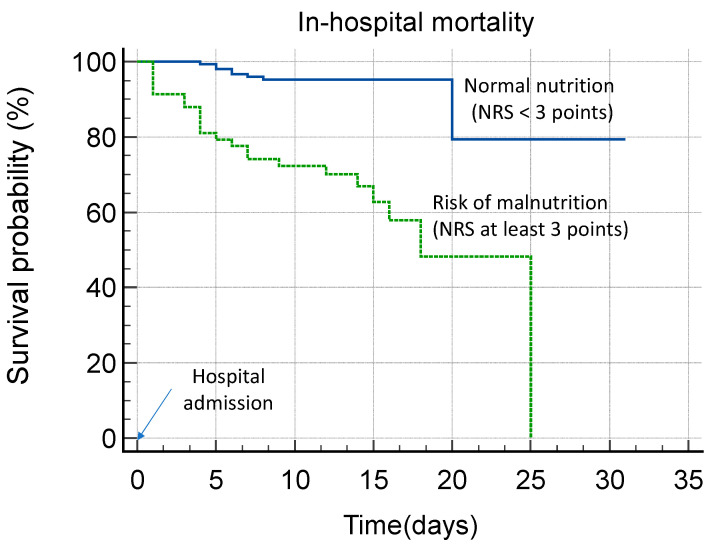
Kaplan–Meier curves presenting the in-hospital mortality rate in COVID-19 patients with increased risk of malnutrition at admission and those with normal nutrition status.

Thus, patients with an NRS ≥ 3, regardless of other variables, had a significantly higher risk of in-hospital mortality compared to those with normal nutritional status (Figure 1).

## 4. Discussion

We investigated the impact of the risk of malnutrition on in-hospital mortality among adult patients with COVID-19 during Delta variant predominance. The risk of malnutrition was significantly associated with an increased risk of mortality, and this association remained independent of factors such as activities of daily living at admission, heightened risk of pressure sores, cognitive impairment, and elevated procalcitonin concentration and WBC count. Furthermore, our study provides additional evidence that malnutrition screening should be systematically implemented as part of hospital admission protocols, particularly in infectious disease units.

Our findings build upon previous studies by reinforcing the predictive value of the NRS-2002 in COVID-19 outcomes, demonstrating that it remains a critical prognostic tool even in the Delta variant context. While several studies have examined malnutrition in COVID-19 patients, few have specifically analyzed its role during the later pandemic stages, where viral characteristics, healthcare responses, and patient demographics may have shifted. Our study highlights the continued importance of nutritional risk assessment, particularly in an era where COVID-19 is transitioning from a global emergency to an endemic disease [22].

We determined the risk of malnutrition using the NRS-2002 questionnaire at the admission to the hospital due to COVID-19. This tool is commonly used in screening for malnutrition and correlates with a worse prognosis in patients hospitalized for various reasons [23,24], including COVID-19 [25,26,27,28,29]. While some research suggests that malnutrition is not significantly linked to poorer outcomes in COVID-19 patients [9,10], all studies utilizing the NRS-2002 consistently report a strong association between malnutrition risk and adverse outcomes [25,26,27,28,29]. Our findings support such claims—patients with positive malnutrition screening had a higher risk of in-hospital death. The simplicity of the NRS-2002 tool, which any healthcare provider can administer, underscores its value in identifying at-risk patients and potentially improving outcomes through targeted interventions. Given its ease of use and strong predictive capability, NRS-2002 may also be valuable in assessing nutritional risks in other infectious diseases with high hospitalization rates.

In our study, the consistency of malnutrition as a significant predictor of in-hospital mortality across all ten models, with hazard ratios ranging from 3.19 to 5.88 and *p*-values consistently below 0.01, underscores its independent and robust association with poor outcomes. The high Nagelkerke’s R^2^ values (ranging from 0.66 to 0.77) for Cox proportional hazard models indicate that they explain a substantial proportion of the variance in mortality. These results suggest that malnutrition is not merely a marker of illness severity but a critical and modifiable risk factor that contributes independently to mortality. Including diverse covariates, such as admission ADL, pressure sores, impaired consciousness, high procalcitonin, and high WBC, did not diminish the significance of malnutrition, highlighting its persistent impact across varying clinical contexts. This finding supports the broader integration of nutritional risk assessment into standard hospital protocols, ensuring early identification and timely intervention.

While COVID-19 may no longer be as severe a global threat as during the pandemic, our findings have broader implications. COVID-19 can be considered a model of a severe infectious disease with a systemic inflammatory response [30,31]. Future pandemics or large-scale outbreaks of infectious diseases may arise, and our study highlights the critical role of nutritional assessment and its predictive potential. Identifying malnutrition early and implementing nutritional interventions could improve survival not only in COVID-19 but also in other acute infectious diseases that elicit a strong inflammatory response. Since COVID-19 often requires hospitalization and is associated with a severe inflammatory response, our findings may be extrapolated to other severe inflammatory conditions. Malnutrition may similarly influence the course of such diseases, underscoring the broader relevance of our results [32]. Our results emphasize the need for standardized nutritional screening upon hospital admission for all high-risk patients, regardless of the underlying condition. Given that nutritional deficiencies are common in older adults and patients with chronic diseases, proactive nutritional management could play a crucial role in improving outcomes in future healthcare crises. Public health initiatives focused on early nutritional intervention could substantially reduce mortality and morbidity in infectious disease outbreaks.

Another significant issue is that advanced age (≥65 years) was strongly associated with a markedly increased risk of in-hospital mortality in COVID-19 patients. This underscores the importance of addressing nutritional status, particularly in older patients requiring hospitalization. Malnutrition in this population is often a complication of dementia, cognitive impairment, or reduced activities of daily living. Many older individuals live alone despite limited physical and cognitive capacity, which may predispose them to the development of malnutrition and its associated complications. Whether malnutrition is an ominous predictor of imminent mortality in such individuals warrants further investigation. Future research should focus on prospective interventional studies to determine whether early nutritional supplementation could improve survival rates, particularly in older or functionally impaired patients. Currently, despite the development of guidelines for nutritional interventions in patients with COVID-19, a significant gap remains in research evaluating their effectiveness [33,34]. Only a few studies in this area have shown that the implementation of adequate and timely nutritional support during the ICU stay of COVID-19 patients independently contributes to better survival [35,36].

There are some limitations of our study that should be stressed. Firstly, the number of variables included in the Cox proportional hazards model could not exceed four for statistical reasons, and there was a risk of model overfitting. With 31 deaths among 220 patients, including five or more variables in the model might risk models overfitting. This may limit the generalizability of our findings, as the model might perform well in this dataset but may not apply as accurately to other populations. However, our primary aim was to explore potential associations and generate hypotheses rather than establish definitive causal relationships. Future studies with larger sample sizes must validate our results and strengthen the evidence base for nutritional interventions in severe inflammatory diseases and/or other infections. Nonetheless, our study’s strength lies in its methodological rigor, use of multiple statistical models, and real-world applicability of the NRS-2002 tool. Future large-scale studies across multiple centers would help validate our findings and explore the potential benefits of targeted nutritional interventions in reducing mortality.

## 5. Conclusions

Our findings emphasize the crucial role of malnutrition in determining adverse outcomes in hospitalized COVID-19 patients. As an independent predictor of in-hospital mortality, malnutrition screening should be essential to patient assessment during admission to the hospital. The strong association between NRS-2002 scores and mortality risk underscores the importance of integrating standardized nutritional assessments into routine clinical workflows, particularly for critically ill patients. Early identification of malnutrition through systematic screening could enable timely interventions, potentially reducing hospital stays and improving overall patient recovery.

While COVID-19 may no longer dominate healthcare priorities, its impact as a severe inflammatory disease provides valuable insights applicable to future infectious outbreaks and other inflammatory conditions. The findings from our study suggest that malnutrition is not merely a comorbidity but a modifiable risk factor that could influence patient outcomes across a range of acute and chronic diseases. This highlights the need for a paradigm shift in clinical practice, where nutritional status is given the same level of importance as other vital prognostic indicators, such as comorbidities and biomarkers of inflammation. We stress the importance of nutritional status evaluation in clinical practice in the management of patients, particularly older ones, with severe inflammatory responses, potentially improving survival in various clinical scenarios.

Future research should explore whether targeted nutritional interventions—such as early enteral or parenteral nutrition—can mitigate the risks associated with malnutrition in hospitalized patients. Additionally, large-scale, multicenter prospective studies are needed to validate our findings in diverse patient populations and healthcare settings. By expanding research in this area, we can better understand how nutritional support strategies may improve clinical outcomes, not only in COVID-19 but also in other infectious and inflammatory diseases. Furthermore, investigating the cost-effectiveness of routine malnutrition screening and early nutritional interventions could provide valuable data for healthcare policymakers, ultimately contributing to more effective patient care strategies.

## Figures and Tables

**Table 1:** Characteristics of analyzed patients hospitalized due to COVID-19, including in-hospital non-survivors and survivors (*n* = 222).

Variable	Total (*n* = 222)	Non-Survivors (*n* = 31)	Survivors (*n* = 191)	*p*-Value
Gender: male	116 (52.2%)	15 (48.4%)	101 (52.9%)	0.7005
Age: over 65 years	141 (63.5%)	30 (96.8%)	111 (58.1%)	<0.0001
Data from the medical history at admission				
Hypertension	118 (53.1%)	18 (58.1%)	100 (52.4%)	0.5682
Cardiovascular diseases (congestive heart failure, ischemic heart disease, arrhythmia)	64 (28.8%)	11(35.5%)	53 (27.7%)	0.3965
Renal failure	18 (8.1%)	4 (12.9%)	14 (7.39%)	0.2898
Neurodegenerative disease	32 (14.4%)	8 (25.8%)	24 (12.6%)	0.0926
Stroke/TIA	38 (17.1%)	8 (25.8%)	30 (15.7%)	0.1972
Diabetes	59 (26.6%)	11 (35.5%)	48 (25.1%)	0.2727
Asthma or COPD	25 (11.3%)	2 (6.5%)	23 (12.0%)	0.5427
Anti-SARS-CoV-2 vaccination	45 (20.3%)	2 (6.5%)	43 (22.5%)	0.0432
Clinical and laboratory data at admission				
ADL (Katz Index < 5)	116 (52.3%)	29 (93.6%)	87 (45.6%)	<0.0001
Cognitive impairment	67 (30.2%)	22 (71.0%)	45 (23.6%)	<0.0001
Increased risk of malnutrition (NRS ≥ 3)	58 (26.1%)	23 (74.2%)	35 (18.3%)	<0.0001
Increased risk of pressure sores (Norton Scale < 14)	81 (35.5%)	23 (74.2%)	58 (30.4%)	<0.0001
Anemia	77 (34.7%)	15 (48.8%)	62 (32.5%)	0.1037
Increased serum CRP (above 20 mg/L)	177 (79.7%)	28 (90.3%)	149 (78.0%)	0.1492
Increased serum procalcitonin (above 0.5 ng/mL)	43 (19.4%)	18 (58.1%)	25 (13.1%)	<0.0001
Increased WBC count (above 10^3^/μL^3^)	84 (37.8%)	19 (62.3%)	65 (34.0%)	0.0050
Increased serum D-dimer (above 0.5 ng/mL)	200 (90.1%)	30 (96.8%)	170 (89.0%)	0.3268
In hospital therapy				
Antiviral treatment against SARS-CoV-2	109 (49.1%)	12 (38.7%)	97 (50.8%)	0.2478
Antibiotic treatment	116 (52.2%)	19 (61.3%)	97 (50.8%)	0.3342
Heparin in therapeutic doses	33 (14.9%)	5 (16.1%)	28 (14.7%)	0.7887
Oxygen therapy needed	199 (89.6%)	31 (100.0%)	168 (88.0%)	0.0510

**Table 2:** The univariable Cox models’ results for the relationships between selected variables and in-hospital mortality in studied COVID-19 patients.

Analyzed Variables	Hazard Ratio (95% CI)	*p*-Value
Age: at least 65 years old	13.50 (1.35–99.48)	0.0106
Anti-SARS-CoV-2 vaccination	0.26 (0.06–1.08)	0.0635
ADL at admission (Katz Index < 5)	10.4 (2.5–43.9)	0.0015
Cognitive impairment	5.08 (2.31–11.14)	<0.0001
Increased risk of malnutrition (NRS ≥ 3)	7.48 (3.32–16.83)	<0.0001
Increased risk of pressure sores (Notron scale < 14)	4.35 (1.94–9.77)	0.0004
Increased serum procalcitonin (above 0.5 ng/mL)	5.39 (2.55–11.40)	<0.0001
Increased WBC count (above 10^3^/μL^3^)	2.25 (1.05–4.83)	0.0377

**Table 3:** The results of the univariate Cox models adjusted to different covariates showing that the risk of malnutrition based on NRS-2002 (≥3) is a significant predictor of in-hospital mortality across all models.

Variables Included in the Model Additionally to Malnutrition	HR (95% CI)	*p*-Value for Malnutrition	R^2^ (Nagelkerke)	AICc (Corrected Akaike Criterion)	*p*-Value for the Model
B, C, D	5.27 (1.92–14.50)	0.0013	0.71	262	<0.0001
B, C, E	4.04 (1.56–10.46)	0.0041	0.77	245	<0.0001
B, C, F	4.44 (1.67–11.83)	0.0029	0.70	254	<0.0001
B, D, E	3.19 (1.36–7.48)	0.0076	0.77	245	<0.0001
B, D, F	3.38 (1.42–8.02)	0.0057	0.71	252	<0.0001
C, D, E	5.31 (1.87–15.12)	0.0017	0.75	248	<0.0001
C, D, F	5.88 (1.99–17.38)	0.0014	0.66	257	<0.0001
B, E, F	3.53 (1.50–8.26)	0.0037	0.77	245	<0.0001
C, E, F	4.97 (1.85–13.36)	0.0015	0.72	251	<0.0001
D, E, F	4.48 (1.89–10.64)	0.0007	0.75	247	<0.0001

Variables included in the model additionally to malnutrition: Covariate B: ADL at admission (Katz Index < 5); Covariate C: Increased risk of pressure sores (Notron scale < 14); Covariate D: Cognitive impairment at admission; Covariate E: Increased serum procalcitonin (above 0.5 ng/mL); Covariate F: Increased WBC count (above 10^3^/μL).

## Data Availability

The datasets used and/or analyzed during the current study are available from the corresponding author upon reasonable request. The data are not publicly available due to being a part of an ongoing study.

## Abbreviations

The following abbreviations are used in this manuscript:

DASH	Dietary Approaches to Stop Hypertension
IUC	Intensive Care Unit
NRS-2002	Nutritional Risk Score 2002
COPD	chronic obstructive pulmonary disease
ADL	Activities of Daily Living
CRP	C-reactive protein
WBC	white blood cells
PCT	procalcitonin
HR	hazard ratio
CI	confidence interval
AICc	corrected Akaike criterion
